# Based on cross-scale fusion attention mechanism network for semantic segmentation for street scenes

**DOI:** 10.3389/fnbot.2023.1204418

**Published:** 2023-08-31

**Authors:** Xin Ye, Lang Gao, Jichen Chen, Mingyue Lei

**Affiliations:** ^1^Institute of Artificial Intelligence and Data Science, Xi'an Technological University, Xi'an, China; ^2^Computer Part III, Xi'an Microelectronics Technology Institute, Xi'an, China

**Keywords:** computer vision, semantic segmentation, channel attention mechanism, residual block, dilation convolution, factorized convolution

## Abstract

Semantic segmentation, which is a fundamental task in computer vision. Every pixel will have a specific semantic class assigned to it through semantic segmentation methods. Embedded systems and mobile devices are difficult to deploy high-accuracy segmentation algorithms. Despite the rapid development of semantic segmentation, the balance between speed and accuracy must be improved. As a solution to the above problems, we created a cross-scale fusion attention mechanism network called CFANet, which fuses feature maps from different scales. We first design a novel efficient residual module (ERM), which applies both dilation convolution and factorized convolution. Our CFANet is mainly constructed from ERM. Subsequently, we designed a new multi-branch channel attention mechanism (MCAM) to refine the feature maps at different levels. Experiment results show that CFANet achieved 70.6% mean intersection over union (mIoU) and 67.7% mIoU on Cityscapes and CamVid datasets, respectively, with inference speeds of 118 FPS and 105 FPS on NVIDIA RTX2080Ti GPU cards with 0.84M parameters.

## Introduction

Semantic segmentation is a computer vision task that involves assigning a label to every pixel for a given image based on its content. In the context of street scenes, this task involves identifying and labeling various objects such as buildings, roads, vehicles, and pedestrians.

In the last 10 years, scene understanding has advanced quickly in the fields of computer vision and photogrammetry, particularly the essential task of semantic segmentation (Yang et al., 2021). Semantic segmentation aims to assign a label for each pixel of the images. It has a wide range of applications, including scene comprehension, autonomous vehicle and driver assistance, and augmented reality (Lu et al., 2019). Enabling autonomous cars to be environmentally aware so they can drive safely, and machines to intelligently analyze medical images, reducing the workload for doctors and dramatically reducing the time it takes to run diagnostic tests.

The cross-scale fusion attention mechanism network uses a combination of convolutional neural networks (CNNs) and attention mechanisms to perform semantic segmentation. CNNs are used to extract features from images at multiple scales, while attention mechanisms are used to selectively focus on important regions of the image.

The attention mechanism is an effective way to promote accuracy by computing attention maps that indicate which regions of the feature maps are most relevant for the segmentation task. The attention maps are then used to weigh the features from different scales before they are fused together. This helps to ensure that important information from all scales is taken into account during the segmentation process.

In recent years, deep convolutional neural networks (DCNNs) have demonstrated their amazing capabilities for Image classification tasks. Since the FCN (Long et al., 2015) was proposed, which is the pioneer for semantic segmentation, DCNNs have shown their power in the task of semantic segmentation. It has become the mainstream of segmentation approaches. Compared to traditional visual algorithms, DCNNs achieve good results with their end-to-end approach.

Of course, the development of image segmentation technology also has many shortcomings that need to be improved. With the development trend of artificial intelligence, the network model is getting deeper and bigger. As the network deepens, training will become more and more difficult, mainly because of the gradient explosion in the network training process of gradient descent. Some methods have also been used to improve the situation, such as changing weights and normalization. However, with the deepening of the network model, the training error increases rather than decreases. The emergence of residual networks solves this problem well, and its performance is greatly improved compared to a traditional network.

Most of the prior networks (Long et al., 2015; Badrinarayanan et al., 2017; Chen et al., 2017) neglected the segmentation efficiency while generating outstanding results. They have several disadvantages, including large storage overhead and low computing efficiency. Specifically, they have high computational and storage requirements. Therefore, creating lightweight and efficient networks to solve the above problems is a major trend. The core of our CFANet is ERM with dilated factorized convolution, which can extract features while keeping the computation requirements low. Our main contributions can be summarized as follows:

a) An ERM, which consists of convolutional decomposition and channel shuffling operations, is designed to extract semantic information while keeping the computational cost low.

b) MCAM is introduced to refine the feature maps at different levels.

c) We achieve 70.6% mIoU and 67.7% mIoU on the Cityscapes and CamVid datasets, respectively, along with the inference speed of 118 FPS and 105 FPS on an NVIDIA RTX2080Ti GPU card.

Overall, the cross-scale fusion attention mechanism network is an effective approach the semantic segmentation of street scenes. It has been shown to achieve state-of-the-art performance on several benchmark datasets, demonstrating its potential for real-world applications such as autonomous driving and urban planning.

## Materials and methods

In this section, the work related to dilated convolution, factorized convolution and real-time semantic segmentation will be discussed. The following is a general overview of the materials and methods used in the cross-scale fusion attention mechanism network for the semantic segmentation of street scenes:

a) Data Collection: A large dataset of street scenes was collected for training and validation of the neural network. This dataset typically includes high-resolution images and corresponding segmentation masks that label each pixel of the image with the corresponding object or class.

b) Pre-processing: The collected data is pre-processed to prepare it for use in the neural network. This may include resizing the images, normalizing the pixel values, and augmenting the data through techniques such as rotation, flipping, and cropping to increase the size and diversity of the dataset.

c) Network Architecture: The cross-scale fusion attention mechanism network architecture is designed and implemented based on the specific requirements of the semantic segmentation task.

d) Training: The network is trained using the pre-processed data through a process of backpropagation, where the weights of the network are adjusted to minimize the loss function. The training process involves multiple iterations or epochs, where the network is trained on batches of images and corresponding segmentation masks.

e) Evaluation: The performance of the network is evaluated on a separate validation dataset to assess its accuracy and generalization ability. Metrics such as mIoU and pixel accuracy are commonly used to evaluate the performance of the network.

f) Testing: The final step involves using the trained network to perform semantic segmentation on new images in real-world applications. This typically involves feeding the input image through the network and generating a segmentation mask that labels each pixel with the corresponding object or class.

Overall, the materials and methods used in the cross-scale fusion attention mechanism network for semantic segmentation of street scenes involve collecting and pre-processing data, designing and implementing the neural network architecture, training and evaluating the network, and finally testing it in real-world applications.

### Dilated convolution

Dilated convolution is a convolutional neural network operation that enables the receptive field of a convolutional layer to be expanded without increasing the number of parameters. It is commonly used in semantic segmentation tasks where the output needs to preserve fine-grained spatial details. In a traditional convolutional layer, each filter kernel slides over the input feature map with a stride of 1, resulting in a receptive field that grows linearly with the kernel size. Dilated convolution, on the other hand, inserts zeros between the kernel values, effectively increasing the kernel's spacing or dilation rate. This means that the receptive field of the dilated convolutional layer can be increased without increasing the number of parameters.

Dilated convolution is commonly used in deep learning architectures for image analysis, such as in semantic segmentation, where it helps to capture multi-scale features and maintain spatial resolution. It has been shown to improve the performance of neural networks in a variety of computer vision tasks.

For segmented tasks, the feature resolution was decreased due to the consecutive pooling operations or convolution striding. This invariance may have a negative impact on detailed segmentation. To overcome this problem, dilated convolution, which has been proven as an effective way for semantic segmentation tasks. For example, Deeplab (Chen et al., 2017) introduced an atrous spatial pyramid pooling module that applied dilated convolution and pyramid framework to enlarge the receptive field. LedNet (Wang et al., 2019) used dilated convolution in the proposed SS-nbt module to enlarge the efficiency and the accuracy of the residual block. RELAXNet (Liu et al., 2022) applied dilated convolution in the process of the depth separable convolution to compress the module model. All of the above methods demonstrate the effectiveness and lightness of dilated convolution in the segmentation task.

### Factorized convolution

In order to improve the inference speed and ensure the segmentation accuracy, factorized convolution is often used to construct lightweight segmentation networks. Factorized convolution is a technique used in deep learning for reducing the computational cost and memory requirements of CNNs. It involves decomposing a standard convolutional operation into two or more separate convolutions, each with a smaller kernel size.

The idea behind factorized convolution is that a large convolutional kernel can be factorized into smaller kernels that are applied sequentially. This reduces the number of parameters in the network and can speed up computation without sacrificing accuracy.

Factorized convolution has several advantages over standard convolutional layers. First, it reduces the number of parameters in the network, which can reduce overfitting and make training faster. Second, it reduces the computational cost of the network by breaking down the convolution into smaller operations. Finally, factorized convolution can improve accuracy in certain cases by allowing for more efficient and targeted feature extraction.

Factorized convolution is commonly used in mobile and embedded deep learning applications where computational and memory resources are limited. It has been shown to be effective in a variety of computer vision tasks, including image classification, object detection, and semantic segmentation.

There are two kinds of factorized methods often used in lightweight networks. One is factorized the standard 3 × 3 convolution into a stacked 1 × 3 and 3 × 1 convolution, and the other is depth separable convolution that factorized the standard convolution into a depth-wise convolution and point-wise convolution. These two factorized methods can dramatically decrease the amount of the parameters. Many real-time semantic segmentation approaches, including FASSD-Net (Rosas-Arias et al., 2021), MDRNet (Dai et al., 2021), and MSCFNet (Gao et al., 2021) use it to construct efficient networks.

### Attention mechanisms

Attention mechanisms are a technique used in deep learning to selectively focus on certain parts of the input data during the learning process. It was initially introduced in natural language processing for machine translation, but has since been applied to other domains, including computer vision and speech recognition.

For humans, when we look at a picture, we consciously notice the salient areas and ignore the less important ones. We ask the computer to imitate our behavior, and motivated by this observation, attention mechanisms are introduced into computer vision in order to imitate this aspect of the human visual system. This is the so-called attention mechanism, which is essentially a mechanism for focusing local information. Attention mechanisms have achieved great success in many visual tasks, including image classification, object detection, semantic segmentation, etc.

The idea behind attention mechanisms is to selectively emphasize different parts of the input data, based on their relevance to the task at hand. This is achieved by assigning a weight to each input element, which determines its relative importance. The weights are learned through the training process, allowing the model to adapt to different input patterns. Attention mechanisms are commonly used in neural networks that process sequential or spatial data, such as recurrent neural networks (RNNs) and CNNs. In RNNs, the attention mechanism is typically used to selectively weight different time steps of the input sequence, while in CNNs, it is used to weight different spatial locations in the feature maps. Attention mechanisms have been shown to improve the performance of neural networks in a variety of tasks, including image captioning, machine translation, and speech recognition. It has become a standard component in many state-of-the-art deep learning architectures.

The channel attention mechanism and the spatial attention mechanism are two often used mechanisms. The purpose of using the channel attention module is to make the input image more meaningful. The importance of each channel of the input image is calculated through the network. So as to achieve the purpose of improving the feature representation ability. The attention mechanism (Vaswani et al., 2017) was originally proposed in the natural language field and it assigns each word a different weight. Now, it has been widely used in computer vision tasks. SENet (Hu et al., 2018) generated the feature map weights by modeling the relationship between channels. Besides the channel attention mechanism, CBAM (Woo et al., 2018) used spatial attention mechanisms to assign weights for pixels. The fusion of the high-level and low-level features in the segmentation tasks is an efficient way to improve the accuracy performance. SaNet (Fan and Ling, 2017) introduced a channel shuffle operation for the fusion of the different level features. JPANet (Hu et al., 2022) presented a bilateral path to fuse the feature from different levels.

## Methodology

In this section, we first introduce our ERM, which is used for feature extraction.

Subsequently, MCAM is proposed by us. Next, we present the MCAM module that includes the attention mechanism, which is used to fuse features at different levels. At the end of this section, we will discuss the overall architecture of our CFANet, which fuses different levels of features.

### Efficient residual module

**Figure 1:**
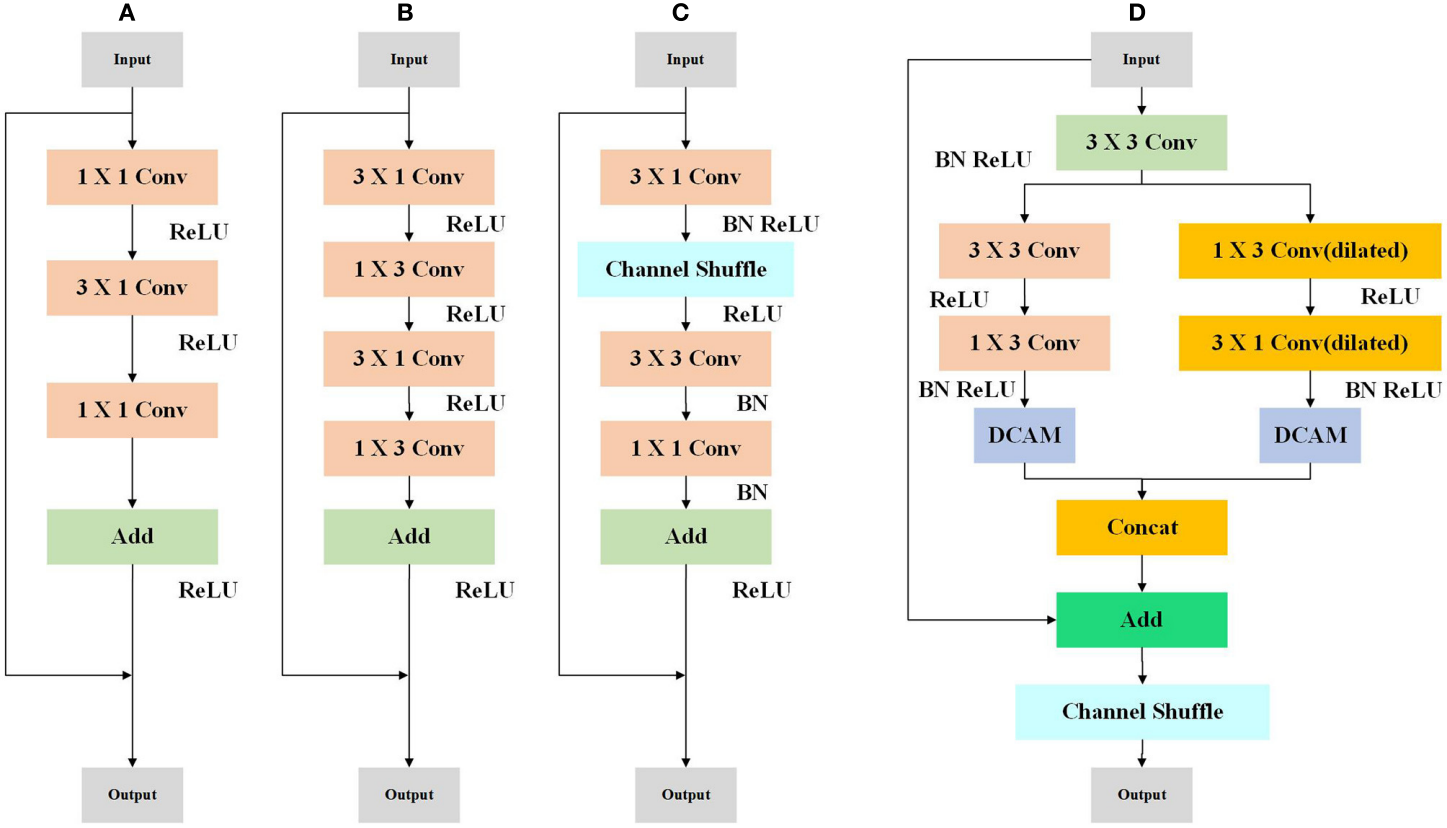
Illustration of the bottleneck **(A)**, non-bottle-1d **(B)**, Shufflenet module **(C)**, and ERM **(D)** residual structure.

We concentrate on enhancing the residual structure's effectiveness, which is frequently used in modern CNNs for computer vision tasks. Recent years have seen numerous successful uses of lightweight residual structures, including bottleneck (Figure 1A), non-bottle-1d (Lu et al., 2019) (Figure 1B), and Shufflenet module (Long et al., 2015) (Figure 1C), motivated by LedNet (Wang et al., 2019) and MSCFNet (Gao et al., 2021), We devise an ERM to improve performance with the limitation of computational capacity. Our ERM module is shown in Figure 1D.

In Figure 1, at the beginning of ERM, a standard 3 × 3 convolution is used to decrease the number of the channel by half. The following is a two-branch structure with depth-wise convolution. To be specific, a standard 3 × 3 is divided into consecutive 1 × 3 and 3 × 1 convolutions. The other branch applies dilated depth-wise convolution, which can help enlarge the receptive field. The two-branch is refined by MCAM, which will be introduced in the next subsection.

### Multi-branch channel attention mechanism

**Figure 2:**
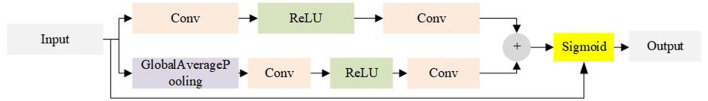
The structure of the MCAM.

The attention mechanism can give varying weights to the traits to draw attention to the crucial ones and ignore the unimportant ones. In this paper, we present MCAM to generate different weights for the channels, which is shown in Figure 2.

The convolution is chosen as the local channel context aggregator, which utilizes point-level channel interactions only for each spatial location. As Figure 2 shows, our MCAM module uses global average pooling and 3 × 3 standard convolution in the upper and bottom branches simultaneously. The results from two branches are added element by element. After that, the sigmoid function is used to generate different weights for channels. This procedure can be expressed as follows:


(1) $$\begin{array}{l} MCAM\left(F\right)=F*\sigma \left(Add\left(AvgP\left(F\right)+Conv^{3\times 3}\left(F\right)\right)\right) \end{array}$$

Where F∈*RC*×*H*×*W* denotes the input feature maps, C, H, W represent the channel, height, and width of the feature map, respectively. σ is sigmoid activation function. Conv^3 × 3^ denotes standard convolution with kernel 3 × 3. Add means the channel wise addition. AvgP is the average pooling operation.

### Network architecture design

**Figure 3:**
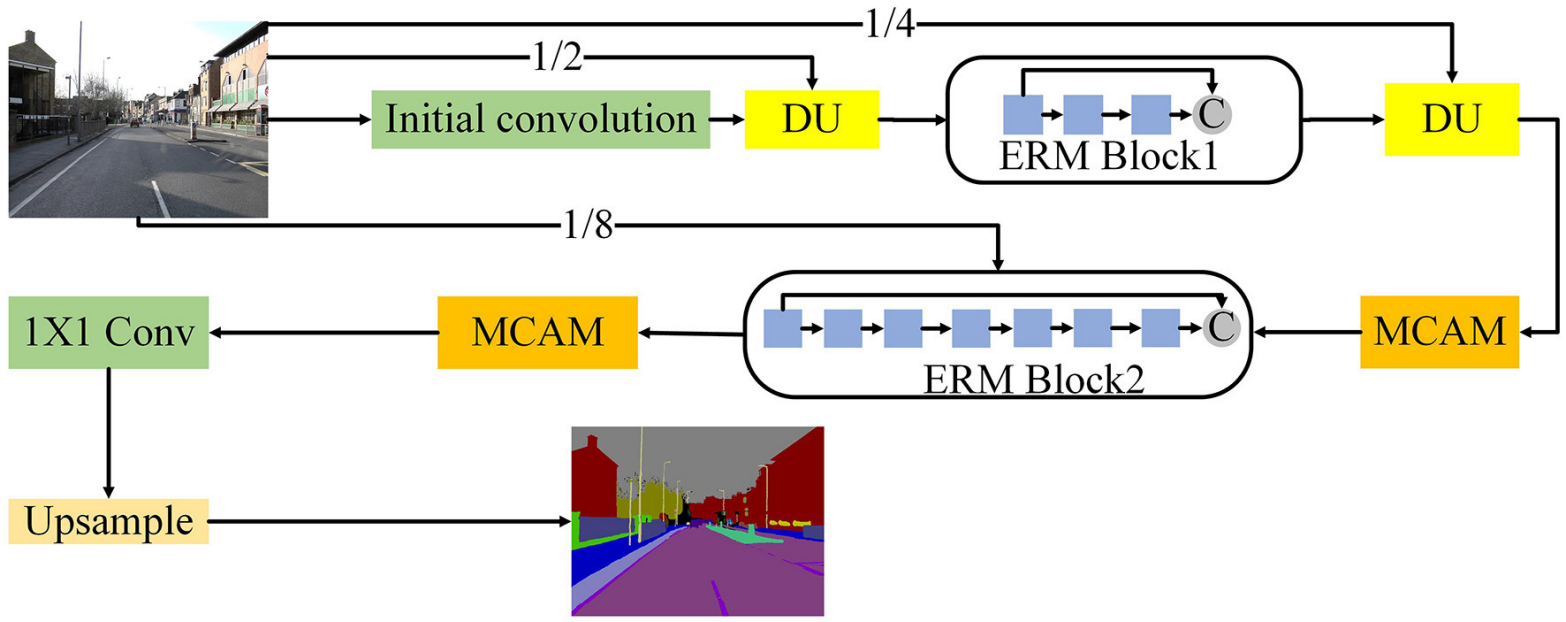
Illustration of the overall architecture of the CFANet.

Based on ERM, we design the architecture of CFANet as shown in Figure 3. In this section, we will introduce the final model of the CFANet.

**Figure 4:**
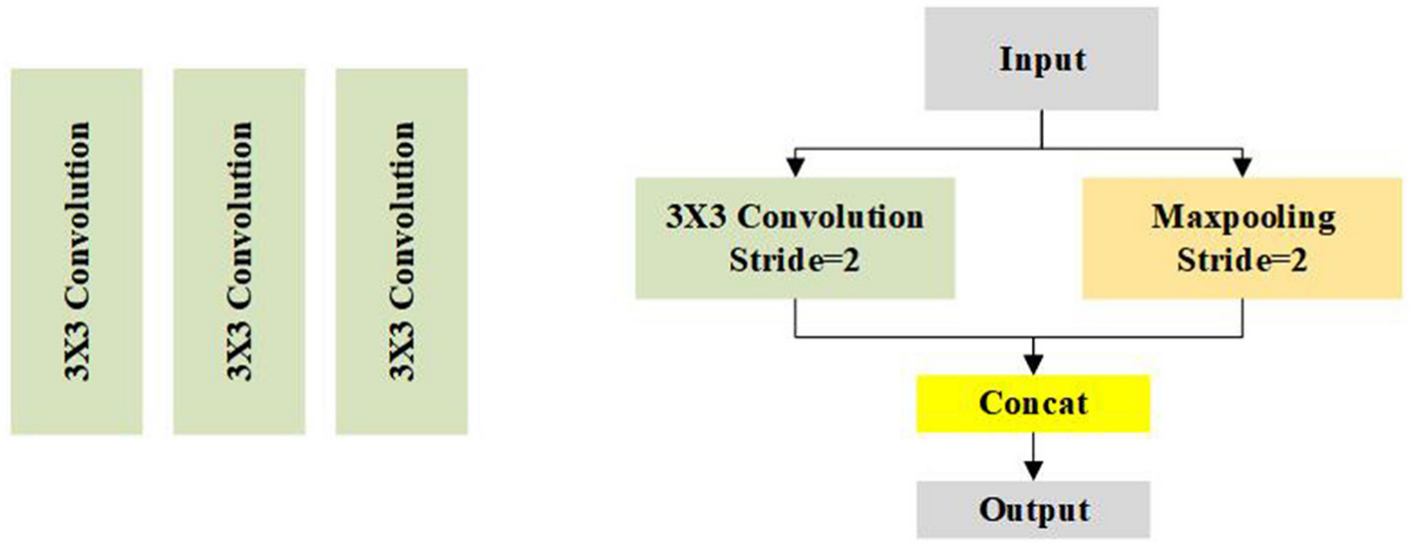
Illustration of the initial convolution and down sampling unit.

As can be seen from Figure 3, we first use three 3 × 3 conservative standard convolutions with stride 2 to extract the initial feature of the input images. After the initial convolution, a down sampling unit is used to reduce the size of the feature map and expand the reception domain. However, too many down sampling operations will cause the information, thus, we only employ three down sampling units in our method, thus, the final resolution of the feature map is 1/8 of the input. Our initial convolution and down sampling unit are shown in Figure 4.

The pseudonym code of our CFANet is shown as follows:

**Algorithm 1:**
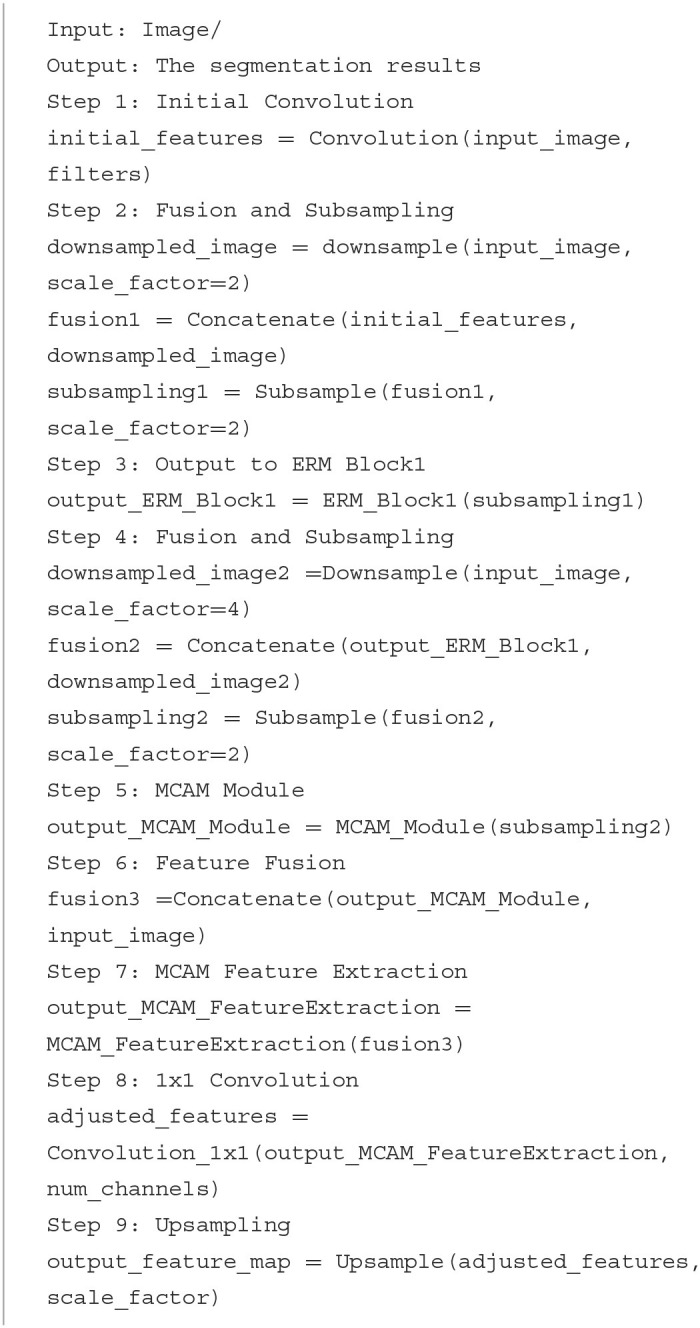
Cross-scale fusion attention net (CFA-Net).

## Experiments

In this part, details and results of our experiments will be presented on the popular semantic segmentation benchmarks Cityscape (Cordts et al., 2016) and CamVid (Brostow et al., 2009). The network was trained on these two data sets, which consisted of high-resolution street view images labeled with pixel-level semantic labels. They used cross-entropy loss functions to train the network and data enhancement techniques such as random scaling and clipping to increase the diversity of the training data. The performance of the proposed network is evaluated using several metrics, including mIoU and pixel accuracy. The results show that the proposed network outperforms several state-of-the-art semantic segmentation networks on the Cityscapes dataset, demonstrating the effectiveness of the cross-scale fusion attention mechanism.

### Datasets

#### Cityscapes dataset

**Figure 5:**
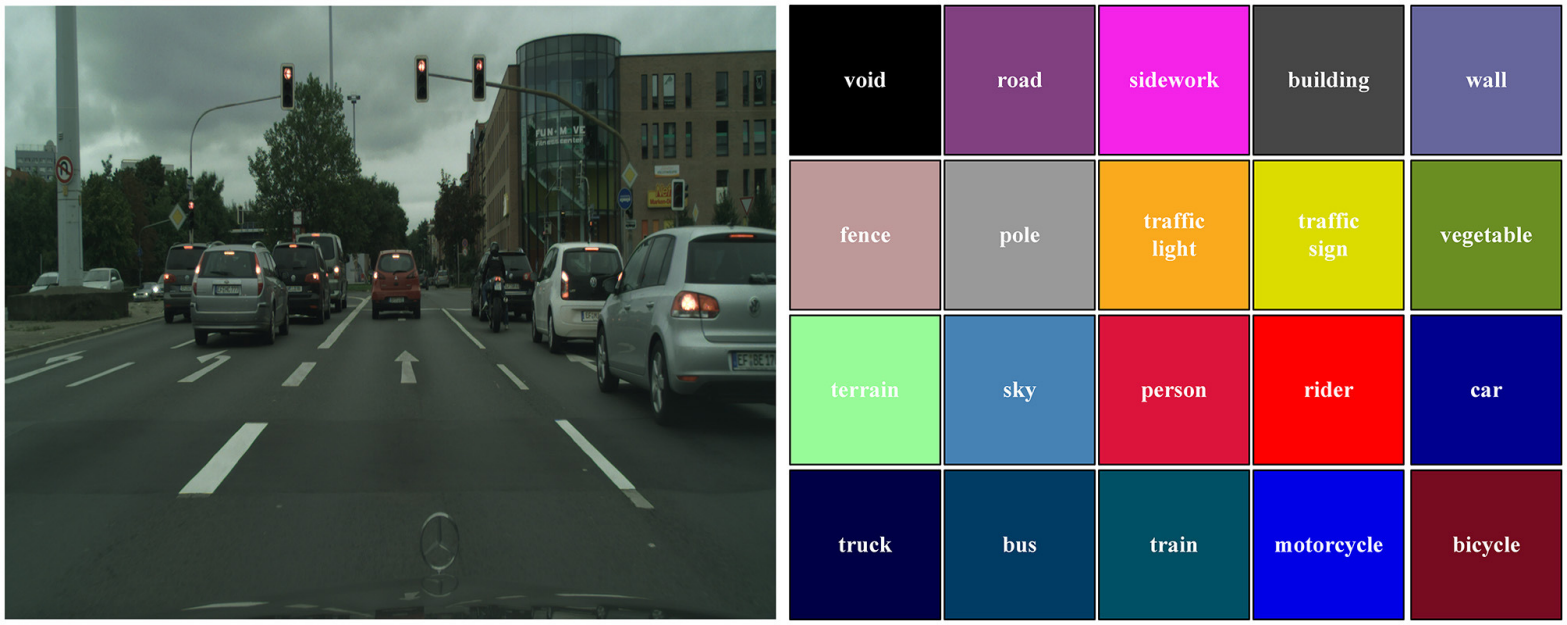
The corresponding images and labels of Cityscapes dataset.

The Cityscapes dataset, contains 19 semantic classes and includes 5,000 fine-labeled samples with the resolution 2,048 × 1,024. The total 5,000 images are divided into training, validation, and test parts. The training parts contain 2,975 images, the validation subset has 500 samples and the test sets have 1,525 images. The sample image and corresponding labels can be seen in Figure 5.

#### CamVid dataset

**Figure 6:**
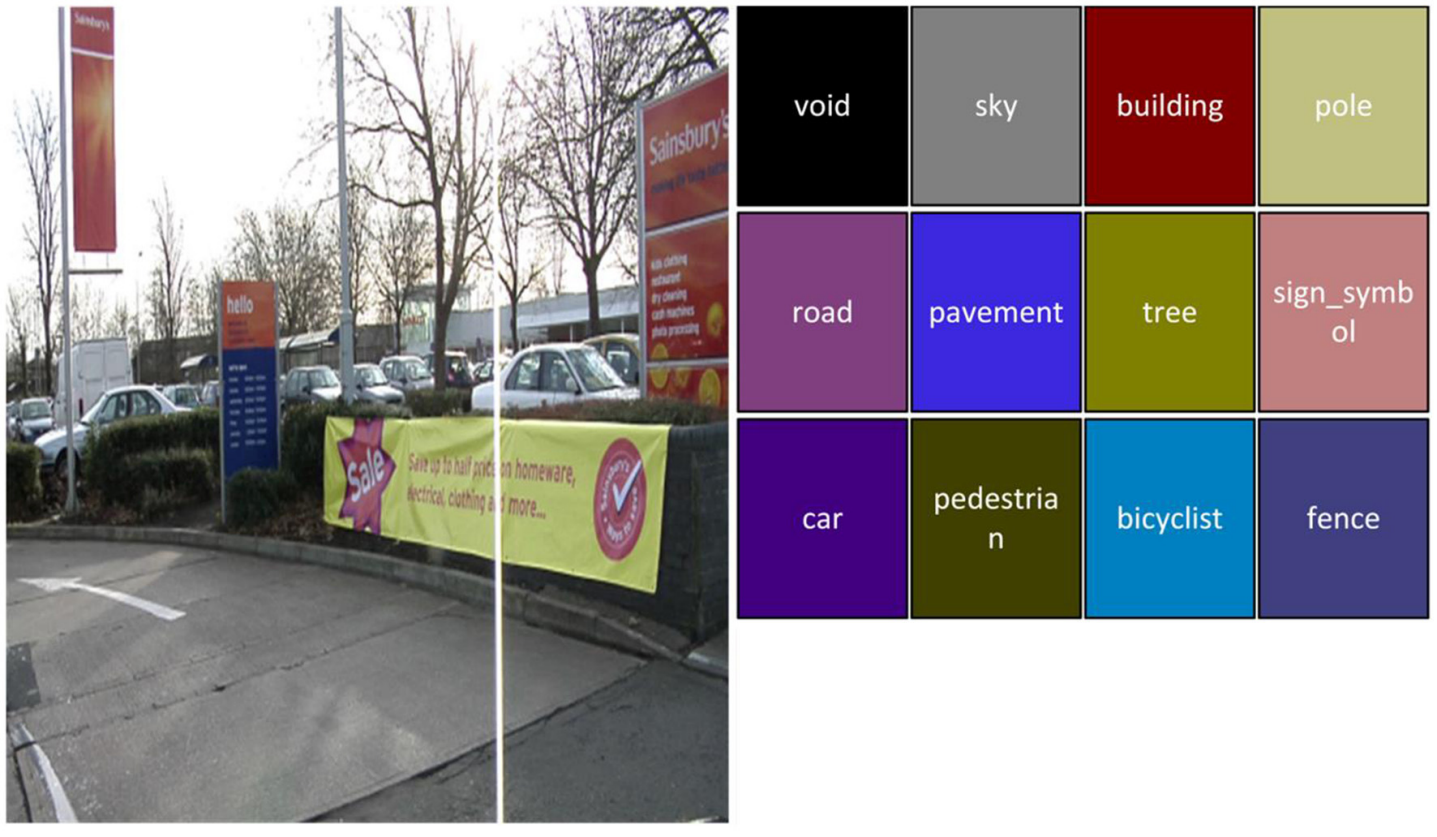
The corresponding images and labels of CamVid dataset.

The CamVid dataset is collected from a car video sequence, which contains 11 semantic classes and includes 710 labeled images (367 images for training, 101 images for validation, and 233 images for testing). The sample image can be seen in Figure 6.

### Data augmentations

In order to overcome the over fitting issue, data enhancement was performed using a horizontal flip and random scale 126. The random scale contains {0.5, 0.75, 1.0, 1.25, 1.5, 1.75, 2.0} Besides, we also use color jitter to adjust the brightness, control, and saturation of the training images and labels.

### Training protocols

We train our network with Stochastic Gradient Descent (Bottou, 2010) (SGD) optimizer on Cityscapes dataset with a batch size of 8 on a single NVIDIA RTX2080Ti Card which has 24 GB GPU memory. The learning rate is adjusted by a polynomial policy in the training process. The polynomial policy is computed by $Ircur=init<uscore>Ir\;\times \;{\left(1-\frac{epoch}{total<uscore>epoch}\right)}^{power}$. The initial learning rate is 4e-2.

When performing training on the CamVid dataset, Adam (Kingma and Ba, 2014) is used as the optimizer with a batch size of 8 and an initial learning rate of 1e-3. We also use a polynomial policy to adjust the learning rate of the training process.

### Ablation studies

In this section, the effectiveness of our proposed MCAM was verified by ablation studies. All the ablation experiments are performed on the CamVid dataset, which training is time-saving. We trained 1,000 epochs for all the ablation experiments.

#### Ablation studies on MCAM

In order to prove the effectiveness of MCAM, we removed all the MCAM in our CFANet. The experiment results can be seen in Table 1.

**Table 1:** Ablation results on MCAM.

**Methods**	**MCAM**	**Paramets (*M*)**	**mIou**
CFANet	√	0.84	67.7
CFANet	×	0.77	66.7

From Table 1, it can be observed that the mIoU decreases by 1% when MCAM is removed. The parameters are reduced to 0.07 million. In other words, our ECAM can effectively increase accuracy with negligible parameters.

### Performance

In this subsection, Compare our algorithm with the state-of-the-art model. We first report the comparison results on Cityscapes and Camvid benchmarks, then analyze the speed of our model and compute the FPS of other state-of-the-art methods under the same status for fair comparison.

#### Performance on Cityscapes datasets

A quantitative and quantitative comparison of the urban landscape with other methods is shown. The comparison metrics consist of input size, backbone network, parameter amount, Flops, and the mIoU, the results can be seen in Table2.

**Table 2:** The comprehensive comparisons on Cityscapes dataset.

**Method**	**Input**	**Backbone**	**Parameters (*M*)**	**FLOPs (*G*)**	**mIoU (%)**
SegNet (Badrinarayanan et al., 2017)	640 × 380	VGG16	29.50	286	57.0
Enet (Paszke et al., 2016)	512 × 1,024	No	0.36	3.8	58.3
SQNet (Hu et al., 2018)	1,024 × 2,048	SqueezeNet	–	270	59.8
ESPNet (Mehta et al., 2018)	512 × 1,024	ESPNet	0.36	113	60.3
CGNet (Wu et al., 2020)	360 × 640	No	0.5	-	64.8
ContextNet (Han et al., 2020)	1,024 × 2,048	No	0.85	–	66.1
EDANet (Yang and Gao, 2019)	512 × 1,024	No	0.68	81	67.3
ERFNet (Romera et al., 2017)	512 × 1,024	No	2.10	–	68.0
Fast-SCNN (Zhang et al., 2018)	1,024 × 2,048	No	1.11	–	68.0
BiseNet (Yu et al., 2018)	768 × 1,536	Xception39	5.80	14.8	68.4
ICNet (Zhao et al., 2017)	2,048 × 1,024	PSPNet	26.50	28.3	69.5
DFANet (Li et al., 2019a)	1,024 × 1,024	Xception	7.80	3.4	71.3
Ours	1,024 × 512	No	0.84	10.4	70.6

**Figure 7:**
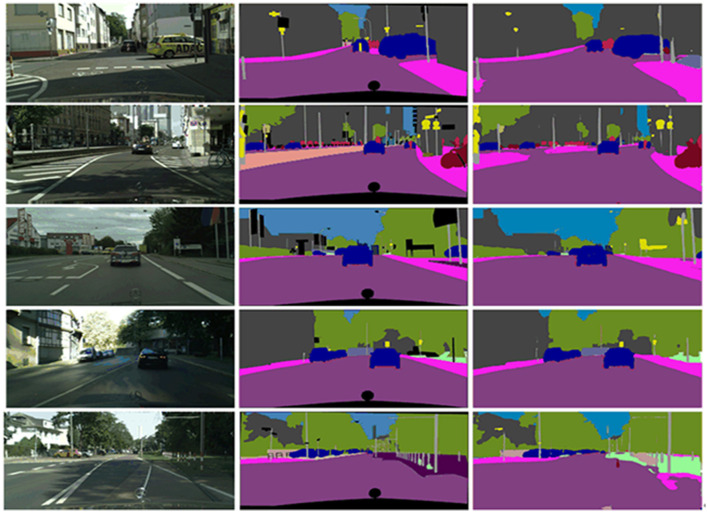
The visual results on Cityscapes validation set (from the most-left to right-most is: input, DFANet, and ours).

It can be observed from Table 2, that the mIoU is comparable to the current state-of-the-art methods, but our CFANet is more lightweight and efficient. The results on Cityscapes show that our approach achieves 71.5% mIoU with only 0.84 million parameters. Compared to DFANet, our method has a similar accuracy but our method only has 0.84 M parameters. Compared to DFANet, our method has a similar accuracy but our method only has 0.84 M parameters. In addition, in order to visualize the results of different methods in terms of segmentation effects, we provide visual comparisons on the Cityscapes validation set. The visual comparison results can be seen from Figure 7.

We also provide a per-class IoU on Cityscapes datasets. Per-class IoU can be seen in Table 3.

**Table 3:** Per-class IoU(%) performance on the Cityscapes testing set.

**Methods**	**Roa**	**Sky**	**Car**	**Veg**	**Bui**	**Sid**	**Ped**	**Bus**	**Tsi**	**Bic**	**Ter**	**TLi**	**Rid**	**Pol**	**Tra**	**Mot**	**Wal**	**Fen**	**Tru**	**Ter**	**mIoU**
SegNet (Badrinarayanan et al., 2017)	96.4	91.8	89.3	87.0	84.0	73.2	62.8	43.1	45.1	51.9	63.8	39.8	42.8	35.7	44.1	35.8	28.4	29.0	38.1	63.8	57.0
Enet (Paszke et al., 2016)	96.3	90.6	90.6	88.6	75.	74.0	65.5	50.5	44.0	55.4	61.4	34.1	38.4	43.4	48.1	38.8	32.2	33.2	36.9	61.4	58 3
ESPNet (Mehta et al., 2018)	97.0	92.6	92.3	90.8	76.2	77.5	67.0	52.5	46.3	57.2	63.2	35.6	40.9	45.0	50.1	41.8	35.0	36.1	38.1	63.2	60.3
CGNet (Wu et al., 2020)	95.5	92.9	90.2	89.6	88.1	78.7	74.9	59.5	63.9	60.2	67.6	59.8	54.9	54.1	25.2	47.3	40.0	43.0	44.1	67.6	64.8
ESPNet-v2 (Mehta et al., 2019)	53.0	42.1	43.5	44.2	53.2	49.3	53.1	52.6	66.8	59.9	60.0	65.9	72.9	78.6	88.8	90.5	91.8	93.3	97.3	60.0	66.2
EDANet (Yang and Gao, 2019)	40.9	46.0	42.0	50.4	56.0	52.3	54.3	59.8	68.7	64.0	65.0	58.7	75.7	80.6	89.5	91.4	92.8	94.2	97.7	65.0	67.3
ERFNet (Romera et al., 2017)	97 7	94 2	92 8	91 4	89 8	81 0	76 8	60 1	65 3	61 7	68 2	59 8	57 1	56 3	51 8	47 3	42 5	48 0	50 8	68 2	68 0
ICNet (Zhao et al., 2017)	97.1	93.5	92.6	91.5	89.7	79.2	74.6	72.7	63.4	70.5	8.3	60.4	56.1	61.5	51.3	53.6	43.2	48.9	51.3	8.3	69.5
DABNet (Li et al., 2019b)	97.9	92.8	93.7	91.8	90.6	82.0	78.1	63.7	67.7	66.8	70.1	63.5	57.8	59.3	56.0	51.3	45.5	50.1	52.8	70.1	70.1
LEDNet (Wang et al., 2019)	98.1	94.9	90.9	92.6	91.6	79.5	76.2	64.0	72.8	71.6	61.2	61.3	53.7	62.8	52.7	44.4	47.7	49.9	64.4	61.2	70.6
EdgeNet (Dourado et al., 2020)	98.1	94.9	94.3	92.4	91.6	83.1	80.4	60.9	71.4	67.7	69.7	67.2	61.1	62.6	52.5	55.3	45.4	50.6	50.0	69.7	71.0
Ours	97.2	81.8	90.1	54.1	55.3	59.8	61.8	71.7	92.2	62.2	92.9	77.5	54.2	92.8	52.3	67.1	55.1	51.2	72.1	92.9	70.6

#### Performance on camvid

To further verify the effectiveness of our CFANet, we also evaluated our CFANet on the CamVid dataset. As shown in Table 4, our CFANet obtained remarkable performance against other methods.

**Table 4:** Comparisons with some of state-of-art methods on CamVid test set.

**Methods**	**Input size**	**Backbone**	**Parameter**	**mIoU**
ENet (Paszke et al., 2016)	360 × 480	No	0.36 M	51.3
SegNet (Badrinarayanan et al., 2017)	360 × 480	VGG16	29.5	55.6
NDNet (Yang et al., 2020)	360 × 480	No	0.5	57.2
DFANet (Li et al., 2019a)	720 × 960	Xception	7.8	64.7
Dilation (Rosas-Arias et al., 2021)	720 × 960	VGG16	140.8	65.3
CGNet (Wu et al., 2020)	360 × 480	No	0.5	65.6
BiseNet (Yu et al., 2018)	720 × 960	Xception39	5.8	65.6
DABNet (Li et al., 2019b)	360 × 480	No	0.76	66.4
FDDWNet (Liu et al., 2019)	360 × 480	No	0.80	66.9
ICNet (Zhao et al., 2017)	720 × 960	PSPNet50	26.5	67.1
Ours(CFANet)	360 × 480	No	0.84	67.7

**Figure 8:**
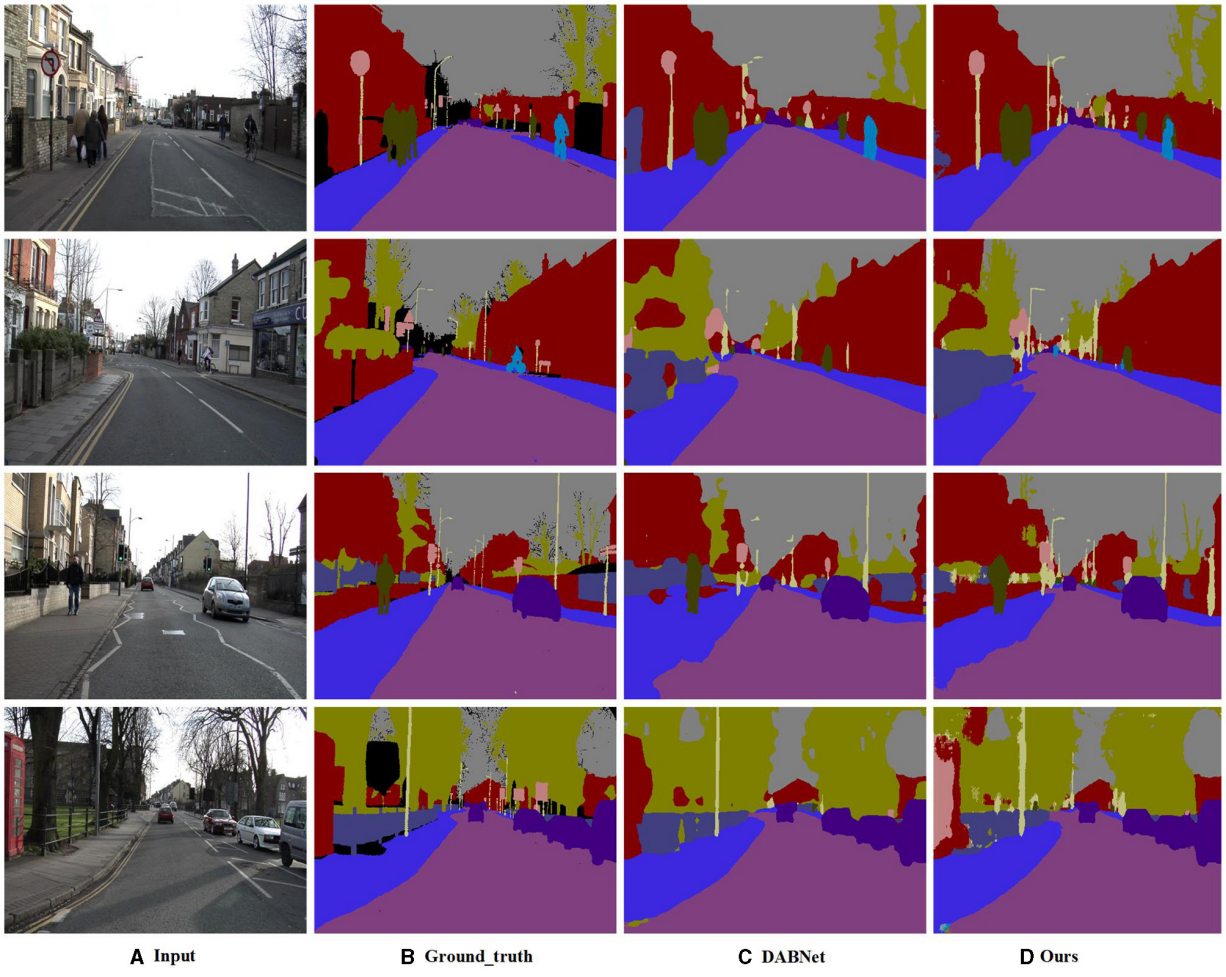
The visual results on Camvid testing set. From the most-left to right-most is: Input **(A)**, Ground-Truth **(B)**, DABNet **(C)**, and ours **(D)**.

From a comprehensive, we select some methods and compared them from four perspectives: input size, backbone, parameter, and mIoU(on test set). As Table 4 shows, our CFANet achieves the best mIoU without backbone. Compared to BiseNet and ICNet, our CFANet is 0.6% higher than ICNet. However, it should be noticed that ICNet has a huge parameter. We provide the visual comparison results of these methods on the CamVid test dataset in Figure 8.

We make a series of supplementary experiments to assess the time performance on an NVIDIA Jeston TX2 platform. The experiment results are shown in Table 5.

**Table 5:** The time performance on NVIDIA Jeston TX2.

**Method**	**Input**	**Platform**	**FPS**	**Accuracy mIoU (%)**
SegNet (Badrinarayanan et al., 2017)	640 × 480	TX2	5	58
Enet (Paszke et al., 2016)	640 × 480	TX2	26	58.3
EDANe (Yang and Gao, 2019)	640 × 480	TX2	42	67.3
ERFNet (Romera et al., 2017)	640 × 480	TX2	39	68.0
Fast-SCNN (Zhang et al., 2018)	640 × 480	TX2	57	68.0
Ours(CFANet)	640 × 480	TX2	55	70.6

A clear comparison is made with other popular algorithms in terms of FLPOS and memory. The results are shown in Table 6.

**Table 6:** The comparison results in terms of FLOPS and amount of memory.

**Method**	**Input**	**Amount of the memory (MB)**	**FLOPs (G)**	**Accuracy mIoU (%)**
SegNet (Badrinarayanan et al., 2017)	512 × 1,024	1,830	326.26	58
Enet (Paszke et al., 2016)	512 × 1,024	0.36	3.8	58.3
SQNet (Hu et al., 2018)	512 × 1,024	895	270	59.8
ESPNet (Mehta et al., 2018)	512 × 1,024	85	3.2	60.3
CGNet (Wu et al., 2020)	360 × 640	783	6.98	64.8
ContextNet (Han et al., 2020)	512 × 1,024	356	1.78	66.1
EDANet (Yang and Gao, 2019)	512 × 1,024	353	8.95	67.3
ERFNet (Romera et al., 2017)	512 × 1,024	806	25.8	68.0
Fast-SCNN (Zhang et al., 2018)	512 × 1,024	309	1.76	68.0
Ours (CFANet)	512 × 1,024	821	10.4	70.6

As shown in Table 6, the memory cost of our CFANet is similar to the ERFNet, but the accuracy performance of our CFANet (in terms of mIoU) is 2.6% higher than it. When compared to the EDANet, the FLOPs of our method are slightly higher than it, but we achieved a 3.3% accuracy promotion, which is significant progress. All the mentioned discussion can prove the effectiveness of our proposed CFANet.

## Conclusions

In this paper, A new semantic segmentation method, CFANet, is proposed. Which fuses 1/2, 1/4, 1/8 feature maps of the input images. Subsequently, we present a novel ERM consisting of convolution decomposition and dilated convolution. We build our core architecture by using ERM. Besides, we devise MCAM to refine the feature map from different stages. Experiment results show that our method achieves 70.6 and 67.7% mIoU along with 118 FPS and 108 FPS on a single NVIDIA 2080Ti GPU card.

In spite of this, we still have a lot of issues to resolve in the near future. In existing lightweight segmentation models, much useful information is lost in order to obtain the smallest possible model size without compromising accuracy. There is still an unsatisfactory level of segmentation accuracy. Furthermore, the inference speed is not fast enough to process high-resolution images. Additionally, while semantic segmentation networks are extremely important for edge devices, their power consumption is not adequately addressed in existing research. For this reason, we are exploring a novel architecture for semantic segmentation to improve the trade-off between inference speed, accuracy, and power consumption in the future.

## Data availability statement

The original contributions presented in the study are included in the article/supplementary material, further inquiries can be directed to the corresponding author.

## Author contributions

XY: conceptualization, methodology, formal analysis, data curation, project administration, and funding acquisition. LG: software, validation, and visualization. JC: investigation and writing—original draft preparation. ML: resources and supervision. XY and LG: writing—review and editing. All authors have read and agreed to the published version of the manuscript.

## References

[B1] Badrinarayanan V. Kendall A. Cipolla R. (2017). Segnet: A deep convolutional encoder-decoder architecture for image segmentation. IEEE Trans. Pattern Anal. Mach. Intell. 39, 2481–2495. 10.1109/TPAMI.2016.2644615 28060704

[B2] Bottou L. (2010). “Large scale machine learning with stochastic gradient descent,” in Proceedings of COMPSTAT'2010 (Paris: Springer), 177–186.

[B3] Brostow G. J. Fauqueur J. Cipolla R. (2009). Semantic object classes in video: a high-definition ground truth database. Pattern Recognit. Lett. 30, 88–97. 10.1016/j.patrec.2008.04.005

[B4] Chen L. C. Papandreou G. Kokkinos I. Murphy K. Yuille A. L. (2017). Deeplab: semantic image segmentation with deep convolutional nets, atrous convolution, and fully connected crfs. IEEE Trans. Pattern Anal. Mach. Intell. 40, 834–848. 10.1109/TPAMI.2017.2699184 28463186

[B5] Cordts M. Omran M. Ramos S. Rehfeld T. Enzweiler M. Benenson R.. (2016). “The cityscapes dataset for semantic urban scene understanding,” in Proceedings of the IEEE Conference on Computer Vision and Pattern Recognition (Las Vegas, NV: IEEE), 3213–3223.

[B6] Dai Y. Wang J. Li J. Li J. (2021). MDRNet: a lightweight network for real-time semantic segmentation in street scenes. Assembly Automat. 46, 725–733. 10.1108/AA-06-2021-0078

[B7] Dourado A. de Campos T. E. Kim H. Hilton A (2020). “Edgenet: semantic scene completion from rgb-d image,” in 2020 25th International Conference on Pattern Recognition (ICPR) (Milan), 503–510. 10.1109/ICPR48806.2021.9413252

[B8] Fan H. Ling H. (2017). “Sanet: structure-aware network for visual trackin,” in Proceedings of the IEEE Conference on Computer Vision and Pattern Recognition Workshops (Honolulu, HI: IEEE), 42–49.

[B9] Gao G. Xu G. Yu Y. Xie J. Yang J. Yue D.. (2021). MSCFNet: a lightweight network with multi-scale context fusion for real-time semantic segmentation. IEEE Transact. Intell. Transport. Syst. 23, 25489–25499. 10.1109/TITS.2021.3098355

[B10] Han W. Zhang Z. Zhang Y. Yu J. Chiu C. C. Qin J.. (2020). Contextnet: Improving convolutional neural networks for automatic speech recognition with global context. arXiv. 3610–3614. 10.21437/Interspeech.2020-2059

[B11] Hu J. Shen L. Sun G. (2018). “Squeeze-and-excitation networks,” in Proceedings of the IEEE Conference on Computer Vision and Pattern Recognition (Salt Lake City, UT: IEEE), 7132–7141.

[B12] Hu X. Jing L. Sehar U. (2022). Joint pyramid attention network for real-time semantic segmentation of urban scenes. Appl. Intell. 52, 580–594. 10.1007/s10489-021-02446-8

[B13] Kingma D. P. Ba J. (2014). Adam: A method for stochastic optimization. arXiv [Preprint]. arXiv: 1412.6980. 10.48550/arXiv.1412.6980

[B14] Li G. Yun I. Kim J. Kim J. (2019). Dabnet: depth-wise asymmetric bottleneck for real-time semantic segmentation. arXiv [Preprint]. arXiv: 1907.11357. 10.48550/arXiv.1907.11357

[B15] Li H. Xiong P. Fan H. Sun J. (2019a). “Dfanet: deep feature aggregation for real-time semantic segmentation,” in Proceedings of the IEEE/CVF Conference on Computer Vision and Pattern Recognition (Long Beach, CA: IEEE), 9522–9531.

[B16] Liu J. Xu X. Shi Y. Deng C. Shi M. (2022). RELAXNet: residual efficient learning and attention expected fusion network for real-time semantic segmentation. Neurocomputing 474, 115–127. 10.1016/j.neucom.2021.12.003

[B17] Liu J. Zhou Q. Qiang Y. Kang B. Wu X. Zheng B.. (2019). “FDDWNet: a lightweight convolutional neural network for real-time semantic segmentation,” in Proceedings of the ICASSP 2020-2020 IEEE International Conference on Acoustics, Speech and Signal Processing (ICASSP) (Barcelona: IEEE), 2373–2377.

[B18] Long J. Shelhamer E. Darrell T. (2015). “Fully convolutional networks for semantic segmentation,” in Proceedings of the IEEE Conference on Computer Vision and Pattern Recognition (Boston, MA: IEEE), 3431–3440. 10.1109/TPAMI.2016.2572683 27244717

[B19] Lu H. Liu Q. Tian D. Li Y. Kim H. Serikawa S.. (2019). The cognitive internet of vehicles for autonomous driving. IEEE Netw. 33, 65–73. 10.1109/MNET.2019.1800339

[B20] Mehta S. Rastegari M. Caspi A. Shapiro L. Hajishirzi H. (2018). “Espnet: efficient spatial pyramid of dilated convolutions for semantic segmentation,” in Proceedings of the European Conference on Computer Vision (ECCV) (Munich), 552–568.

[B21] Mehta S. Rastegari M. Shapiro L. Hajishirzi H. (2019). “Espnetv2: a light-weight, power efficient, and general purpose convolu-tional neural network,” in Proceedings of the IEEE/CVF Conference on Computer Vision and Pattern Recognition, 9190–9200.

[B22] Paszke A. Chaurasia A. Kim S. Culurciello E. (2016). Enet: a deep neural network architecture for real-time semantic segmentation. arXiv [Preprint]. arXiv: 1606.02147. 10.48550/arXiv.1606.02147

[B23] Romera E. Alvarez J. M. Bergasa L. M. Arroyo R. (2017). Erfnet: efficient residual factorized convnet for real-time semantic segmentation. IEEE Transact. Intell. Transport. Syst. 19, 263–272. 10.1109/TITS.2017.2750080

[B24] Rosas-Arias L. Benitez-Garcia G. Portillo-Portillo J. Olivares-Mercado J. Sanchez-Perez G. Yanai K.. (2021). FASSD-Net: fast and accurate real-time semantic segmentation for embedded systems. IEEE Transact. Intell. Transport. Syst. 23, 14339–14360. 10.1109/ICPR48806.2021.9413176

[B25] Vaswani A. Shazeer N. Parmar N. Uszkoreit J. Jones L. Gomez A. N.. (2017). Attention is all you need. Adv. Neural Inf. Process. Syst. 30, 6000–6010. 10.5555/3295222.3295349

[B26] Wang Y. Zhou Q. Liu J. Xiong J. Gao G. Wu X.. (2019). “Lednet: a lightweight encoder-decoder network for real-timesemantic segmentation,” in Proceedings of the 2019 IEEE International Conference on Image Processing (ICIP) (Taipei: IEEE), 1860–1864.

[B27] Woo S. Park J. Lee J. Y. Kweon I. S. (2018). “Cbam: convolutional block attention module,” in Proceedings of the European Conference on Computer Vision (ECCV) (Munich), 3–19.

[B28] Wu T. Tang S. Zhang R. Cao J. Zhang Y. (2020). Cgnet: a light-weight context guided network for semantic segmentation. IEEE Transact. Image Process. 30, 1169–1179. 10.1109/TIP.2020.3042065 33306466

[B29] Yang C. Gao F. (2019). “EDA-Net: dense aggregation of deep and shallow information achieves quantitative photoacoustic blood oxygenation imaging deep in human breast,” in Proceedings of the International Conference on Medical Image Computing and Computer-Assisted Intervention (Springer), 246–254.

[B30] Yang M. Y. Kumaar S. Lyu Y. Nex F. (2021). Real-time semantic segmentation with context aggregation network. ISPRS J. Photogr. Remote Sens. 178, 124–134. 10.1016/j.isprsjprs.2021.06.006

[B31] Yang Z. Yu H. Fu Q. Sun W. Jia W. Sun M.. (2020). NDNet: Narrow while deep network for real-time semantic segmentation. IEEE Transact. Intell. Transport. Syst. 22, 5508–5519. 10.1109/TITS.2020.2987816

[B32] Yu C. Wang J. Peng C. Gao C. Yu G. Sang N.. (2018). “Bisenet: bilateral segmentation network for real-time semantic seg-mentation,” in Proceedings of the European Conference on Computer Vision (Munich), 325–341.

[B33] Zhang X. Chen Z. Wu Q. J. Cai L. Lu D. Li X.. (2018). Fast semantic segmentation for scene perception. IEEE Transact. Ind. Informat. 15, 1183–1192. 10.1109/TII.2018.2849348

[B34] Zhao H. Qi X. Shen X. Shi J. Jia J. (2017). “Icnet for real-time semantic segmentation on high-resolution images,” in Proceedings of the European Conference on Computer Vision (ECCV) (Venice), 405–420.

